# Effectiveness of Serious Games for Leap Motion on the Functionality of the Upper Limb in Parkinson's Disease: A Feasibility Study

**DOI:** 10.1155/2018/7148427

**Published:** 2018-04-11

**Authors:** Edwin Daniel Oña, Carlos Balaguer, Roberto Cano-de la Cuerda, Susana Collado-Vázquez, Alberto Jardón

**Affiliations:** ^1^Robotics Lab, University Carlos III of Madrid, Avda. de la Universidad 30, 28911 Leganés, Spain; ^2^Faculty of Health Sciences, King Juan Carlos University, Avda. de Atenas s/n, Alcorcón, Spain

## Abstract

The design and application of Serious Games (SG) based on the Leap Motion sensor are presented as a tool to support the rehabilitation therapies for upper limbs. Initially, the design principles and their implementation are described, focusing on improving both unilateral and bilateral manual dexterity and coordination. The design of the games has been supervised by specialized therapists. To assess the therapeutic effectiveness of the proposed system, a protocol of trials with Parkinson's patients has been defined. Evaluations of the physical condition of the participants in the study, at the beginning and at the end of the treatment, are carried out using standard tests. The specific measurements of each game give the therapist more detailed information about the patients' evolution after finishing the planned protocol. The obtained results support the fact that the set of developed video games can be combined to define different therapy protocols and that the information obtained is richer than the one obtained through current clinical metrics, serving as method of motor function assessment.

## 1. Introduction

Parkinson's disease (PD) is defined as a chronic neurodegenerative disorder caused by the destruction of dopaminergic neurons located at the basal ganglia. These central nervous system (CNS) neurons are used as primary neurotransmitter dopamine, which is responsible for transmitting the necessary information for the correct control of movements [1, 2]. It is considered the most frequent neurodegenerative disease after Alzheimer's disease and the most common movement disorders [3, 4].

PD prevalence and incidence present a marked geographic variation. In the world population, it can be found that 1-2/1000 people suffer the disease [5]. In Europe, a prevalence rate of 1.6% of the total European population is estimated [6, 7]. PD is characterized by a symptomatic tetrad that consists of resting tremor, stiffness, bradykinesia, and alteration of the straightening reflexes [2, 8]. It also presents other symptoms such as decreased facial expression, sialorrhea, arterial hypotension, depression, and cognitive impairment, among others, with the nonmotor symptoms of the disease being important [9]. These symptoms impair the performance of their daily activities, reducing their level of independence [10].

Currently, there is no curative treatment for PD. The treatment focuses on the symptomatology and to prevent the progression of the disease. The drugs currently used are indicated to compensate the dopamine deficit [11]. The most commonly drug used is levodopa, although dopaminergic agonists, catechol-O-methyltransferase (COMT) inhibitors, anticholinergics, and amantadine are also used [2, 12].

However, not only can therapies with specific drugs be improved, as SG have been shown to play an important role. There is scientific evidence about the benefit of rehabilitation treatment in PD [13–15]. In the field of neurorehabilitation, virtual reality (VR) and interactive video games, such as immersive VR devices, are beginning to be accepted as adjunctive therapeutic tools in the treatment of neurological patients, through real-time simulation and multiple sensorial channels, providing the opportunity to perform functional, repetitive, and rewarding activities [16–19]. Commercial video game consoles such as the Nintendo Wii, the Play Station Eye Toy, or Microsoft XBOX with their Kinect device have been quickly adapted in the clinical setting as low cost options in rehabilitation treatment in patients with PD with various studies which support its clinical use.

New devices have appeared on the market as the Leap Motion Controller (LMC), framed within semi-immersive RV equipment that records movement of the patient's upper extremities without the need to place sensors or devices on the body. Thus, a virtual image of the upper limbs can be generated on a computer screen in which the patient will have to perform movements according to the exercises purposed (touching and picking up objects, ordering figures, playing a piano, flipping hands, among others). However, scientific studies are needed to support its therapeutic use in the treatment of motor disorders of the upper limbs in PD, since it is frequent that a wide repertoire of limitations in the development of functional activities appears, as well as restrictions on participation due to alterations of the upper limbs, throughout the progression of the disease.

In this paper, the feasibility of the LMC-based video games as a rehabilitation tool in the PD treatment is studied. For that purpose, a pilot study was conducted at Parkinson's center with patients using a series of LMC-based video games, during a training protocol defined by therapists. In Section 2, related works are exposed. The proposed methodology and the design principles of the games are described in Section 3. The development of the LMC-based video games and the functionality of each one are shown in Section 4. The definition of the treatment protocol and the obtained results are presented in Section 5. The effectiveness of treatment focused on the video games contribution is discussed in Section 6. Finally, the conclusions are summarized in Section 7.

## 2. Related Works

The use of the Leap Motion Controller (LMC) has been extended from its initial purpose in the entertainment industry, towards different applications based on gesture-recognition such as remote control, sign language translation, and augmented reality and also in health care. In healthcare applications, due to the ability to detect with high precision the finger joints and their movements, the LMC has been used in systems oriented to the rehabilitation of fine and gross manual dexterity, enhanced by a virtual environment that stimulates to the patient.

On the one hand, several works focused on hand motor recovery using only the LMC and a virtual environment are found. In [20], the prefrontal cortex haemodynamic responses during the executions of demanding manual tasks performed in a semi-immersive VR environment are studied. The LMC is used to track the hand movements and to enable subjects to transpose their hand movements within a virtual 3D task. In [21], the user-centered methodology for the design of SG based on LMC is presented. The implemented exergame accomplished with both the users and the therapists considerations for the hand rehabilitation. In [22], the Fruit Ninja game was modified to use LMC for the finger individuation training. The results suggest that Fruit Ninja's score is a good indicator of the hand function according to the high correlation with the standard clinical assessment scores such as Fugl-Meyer (FMA) and Box and Blocks Test (BBT). In [23], the LMC as a gesture controlled input device for computer games was studied. The experience with the LMC into two different game setups was evaluated, investigating differences between gamers and nongamers with 15 participants. Results indicated the potential in terms of user engagement and training efforts for short-time experiences. However, the study results also indicated that gesture-based controls are rated as exhausting after about 20 minutes. While the suitability for traditional video games was thus described as limited, users saw potential in gesture-based controls as training and rehabilitation tools.

Thanks to the portability and low cost of the sensor, the LMC is appropriated to perform exercises at home and remotely supervised by clinicians. Thus, for example, a tool for doctor on which they can prescribe patient to imitate standard exercise hand motion and get automatic feedback, such as score, is proposed in [24]. According to similarity in the scoring, the rehabilitation effect is enhanced. Another similar study, but focused on the cerebral palsy treatment, is shown in [25]. Because the purpose of these systems is to measure the similarity between the standard gestures and those performed by the patient, an immersive virtual environment is not necessary. A study for the treatment of motor and cognitive impairments in children with cerebral palsy is addressed in [26]. Integration between patient and virtual environment occurs through the LMC plus the electroencephalographic sensor MindWave, responsible for measuring attention levels during task execution. Based on results, the level of attention can be correlated with the evolution of the clinical condition.

Besides, others studies integrate support devices in addition to the LMC to assist the patient. In [27], the fusion of the LMC and the Omega.7 haptic sensor with force feedback capabilities has enabled a bilateral rehabilitation training therapy. The LMC tracks the healthy hand and the Omega.7 device haptically interacts with the impaired hand. It allows bilateral complementary tasks for the training of the coordinated cooperation of the paretic arm and intact arm. Other assisted rehabilitation systems are addressed in [28], using the LMC to visualize in a virtual environment the feedback forces sent by a 3D-printed hand orthosis. The hand orthosis is also commanded by four servomotors that eases the full development of the proposed tasks.

On the other hand, the LMC not only has been used as a rehabilitation tool, but also has been used to automate the assessment of the functionality of the hand. This issue is addressed in [29], where an automated system based on the Simple Test for Evaluating Hand Function (STEF) was implemented. In the case of the Parkinson treatment, a novel index of finger-tapping severity, called “PDFTsi,” was introduced in [30]. This index quantifies the severity of symptoms related to the finger tapping of PD patients. Several works are focused on the use of LMC to measure the hand tremor. In [31], the authors propose the implementation of an unobtrusively system to detect tremor, using the LCM and the Vuzix M100 smart glasses. Similar work but using only the LMC is studied in [32]. A novel approach of tremor quantification based on an open-source mobile app is presented in [33].

Due to the fact that the integration of LMC technology into healthcare applications has begun to occur rapidly, the validation of the sensor data output [34] and the feasibility in neurorehabilitation [35] are important research goals. The results of these studies provided a proof of concept that LMC can be a suitable tool for videogame-based therapy in hand rehabilitation.

## 3. Material and Methods

The Serious Games (SG) developed for this study try to imitate exercises included on traditional physical therapy, such as palmar prehension, fingers' flexion, and extension or hand pronation-supination, with the added value that the immersive virtual environment tries to hook the patient to the point of not focusing on the fact of being in a rehabilitation session. This rehabilitation method using SG is proposed for patients with limited mobility in order to restore their ability to independently perform the basic activities of daily living (ADL) or to recover a lost or diminished function by performing exercises on a regular basis. To cover these specific objectives, several video games have been created to exercise different purposes proposed by healthcare professionals. These SG not only are beneficial to recover physical mobility, but also favor the perception of visual acuity, whether the subject has it atrophied or not. This means that although the idea of these games is mainly to work at motor level, they also exercise the cognitive and perceptive capacities of the users. Although the study was carried out with patients with PD, the games try to be as less selective as possible with the target public, being able to be particularized considering the injuries and physical conditions of each user. In this way, it has been determined that the games are favorable for subjects with motor limitations due to suffering any of the following pathologies: PD, people who have suffered a stroke, arthritis, osteoarthritis, manual stiffness, wrist and/or fingers fracture, tennis or golfer elbow, and shoulder injuries.

### 3.1. Design Principles

**Figure 1:**
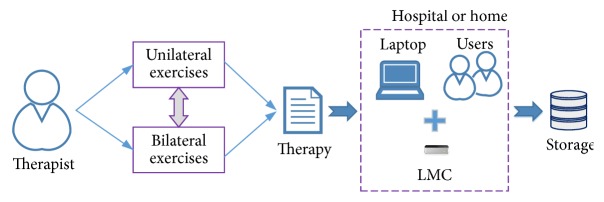
Framework for the upper limb rehabilitation.

In this section, we expose the methods used for the creation of the video games, together with a detailed description of them. The idea was to develop a flexible game platform that allows the clinics to perform the rehabilitation sessions. The video games should include a record of the patients' progress and a minimum set of “how to play” instructions and must be able to give feedback of goals achievement to both patients and therapists. After deep review of LMC sensing capabilities and discussions with occupational therapists, a set of design requirements were chosen to achieve the rehabilitation goals. In Figure 1, the main components of the proposed framework for the development of SG for rehabilitation are described. Then, it was agreed that the implementation of these video games should fulfill the next specifications.

#### 3.1.1. User Interface

It is essential for the interface to allow patients run the video games easily and in an intuitive way, along with simple and clear instructions. For easiness and portability, a simple laptop should be enough to run the games. In the design, it has been noticed that voice instructions complement those shown on screen, so the games count with guide through messages, images, and audios to assist favorably to any type of user. Furthermore, attractive graphics awake interest and help patients to get involved in the exercises. These games try to influence the users' mood while doing rehabilitation by motivating them in a comfortable and innovative virtual environment.

#### 3.1.2. Game Dynamics

The games' sessions ought to be intuitive and straightforward. They are oriented to execute different tasks in which users will be able to perform free articular movements, but a few conditions will be imposed in the way the exercises must be done with the intention that patients are forced to make specific actions and movements which will be part of the therapeutic evaluation. To assure the usability, the games include adjustable features in order to allow physiotherapists make the games suitable for each patient's pathology and conditions. Therapists design the right set of exercises and the sequence of them to be performed by the user, generating the specific treatment protocol scheme as a “recipe” for the specific disease and patient. This is represented in Figure 1 as therapy component.

#### 3.1.3. User's Incentive

As the user performs the unilateral exercises (moving only one arm each time) and bilateral exercises (using both arms) the games save how much time the patient has spent on completing each mission. These results are shown on screen through a bar chart proportional to the time, this way the users can compare how long it has taken to make the exercises with each finger or hand, depending on the game. This system motivates players to improve their times, stimulating their progress during the rehabilitation process.

#### 3.1.4. Clinical Outcomes

An essential outcome to obtain from this rehabilitation through video games is the clinical data to be analyzed by healthcare professionals. Based on therapists' directives, the developed games extract and store information about the human joints' trajectories together with movement ranges during the exercises and the time it takes to perform each game. This recorded data informs about the quality of the exercise performance, the progress of the patient along the sessions, so after its analysis we could conclude about the utility of the virtual therapy.

#### 3.1.5. Automatic Data Store

The information obtained in each session will be automatically stored in the patient's record in a format that medical staff can easily handle to make their evaluations. In this case, CSV files easily match the specifications required and its content can be simply managed. This way, it is possible to access to an updated report of each patient, allowing the physician check remotely the therapy's progress. Each patient record is identified by a code, so their privacy is guaranteed.

#### 3.1.6. Reliable Data Acquisition

Tracking patients' movements is one of the most important issues in order to do a diagnosis or evaluation. Including this data in the generated report allows the therapist to obtain more detailed data to analyze and follow the patient's recovery. The video games technology provides useful way of tracking the patient movements and automatically registers such information, giving support to follow closely the patients' evolution. The idea is to validate if a low cost and portable device, such as LMC, is good enough to develop autonomous tool for “at home” rehabilitation therapies.

### 3.2. Development Tools

The previous Figure 1 includes the main components needed to use the developed SG, mainly a laptop or a PC plus the LMC plugged to its port. Due to this minimum infrastructure, the system could be used everywhere.

#### 3.2.1. Hardware Tools

Leap Motion has been chosen as the most suitable capture instrument for the video games developed due to its portability and low cost; its good precision in the tracking of the different parts of the hand, even its SDK includes functions that facilitate the measurement of the movements and positions of the joints of the fingers and the palm of the hand; its clear results; its ease of use, because thanks to not needing markers for the tracing, it is not intrusive with the patients and it is quick to install. Using the Leap Motion device, interaction with the computer without any physical contact is allowed.

#### 3.2.2. Software Tools

The games were developed using the game engine Unity and C# scripting for the game scripts. This open-source engine allows the video games created to be accessible and free. The source code of the project is hosted by Github in the link, where also several screen-shots are available.

## 4. Games Development

A series of video games focusing on the physical rehabilitation of the upper limbs of patients suffering from some type of motor limitation were designed. According to the requirements and indications from healthcare professionals, six games were developed: Piano (PI), Reach Game (RG), Sequence Game (SG), Grab Game (GG), Pinch Game (PG), and Flip Game (FG); each one of them focused on diverse rehabilitation workout.

**Figure 2:**
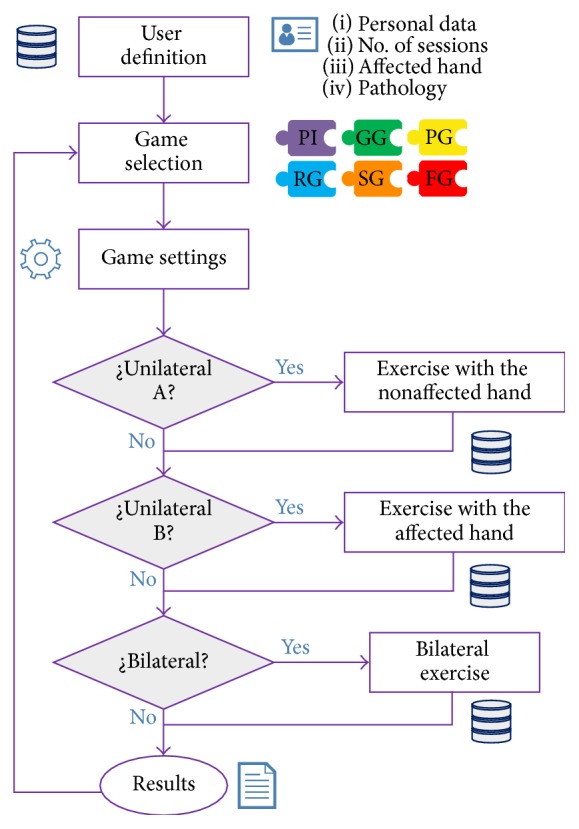
Flowchart of the videogames execution.

Users must follow a set of screens in order to accomplish all the exercises. As showed schematically in Figure 2, the execution of the games is as follows. The first menu screen requests for personal information about the subject, the number of the sessions, which hand is more affected, and what pathology takes the patient to carry out the rehabilitation therapy. If the user is already in the DDBB, after login, a new session identifier is automatically assigned. Once this data is collected, a set of games is available. Then the game follows the defined rehabilitation protocol, understood as the selection of which games, and the proper sequence of games for each session previously defined by the therapist. By default, if no protocol has been defined, the user must select in a menu the game to play from the ones described in next section. After the game activation, when the hands are introduced over the Leap Motion device, they will be virtually represented on screen and patients will be required to move them within the device's area of detection and to perform different gestures to execute the different exercises.

This type of rehabilitation with video games, in contrast to the traditional one, contributes on a motivating context, presenting rich and functional stimuli for the patient. Therefore, these games have been created with the purpose of engaging, thus increasing the active participation of the subject in the rehabilitation program.

### 4.1. Implemented Games

#### 4.1.1. Piano Game (PI)

This game simulates a piano with ten keys, each one corresponding to one finger of each hand. During the game, the highlighted key that is indicated must be pressed by the appropriate finger, keeping the hand open and lowering the finger that will take down the key until it sounds. The keys are highlighted first in order, from the pinkie to the thumb, and then in random sequence. Series will be played in order of each hand and then for both hands simultaneously. It seeks to exercise the dissociation of the fingers by situating each finger over a piano key, stretching them individually downwards, and then recovering the initial position with the hand completely open. These finger movements involve a fine motor unilateral and bilateral coordination and a fine manual dexterity. Note that, along the performance of the game, arm posture control is required, keeping the hand over the Leap Motion device that virtually places the hands on the piano. Furthermore, the game includes a section where the patient must remember a sequence of a certain number of keys that are illuminated and must repeat (after the series shown). This feature adds to the video game the attention and retention training component.

#### 4.1.2. Reach Game (RG)

During this game, the patient's virtual finger must touch the indicated cube among several cubes that appear on screen. As the cubes are reached, they fall to the floor and the next target cube is indicated until the last of them has been dropped. The cubes on the screen are located at different heights and depths. Thus, the sensation of the patients' spatial perception is created, making them move the arms in the space above the LMC device until the correct position of the target cube is found. The highlighted one is the goal to be touched and the rest of them become obstacles to be avoided. The purpose of this exercise is to motivate the users to move the upper limbs of the body to reach the virtual cube, so they have to make specific movements of extension of the fingers, contraction, and stretching of the elbows and abduction and adduction of the shoulders. Also, the subject trains gross motor unilateral and bilateral coordination.

#### 4.1.3. Sequence Game (SG)

In this game, the patient's objective is to memorize the sequence that is reproduced through a color change of the cubes that appear on the screen. At the end of the sequence, the user must repeat it by reaching the cubes in the same order in which they were shown. As in the Reach Game, the physical movements and skills mentioned before are trained, but this game adds the exercising of visual sequential memory.

#### 4.1.4. Grab Game (GG)

The target of this game motivates the patient to perform the movements of closing and opening the hand without resistance. A set of cubes is arranged in a specific layout and a red sphere is shown in the central part of the screen. The user must reach the indicated cube, make the gesture of grip with all the fingers flexed, and then with the fist closed move the grabbed cube to the red sphere and, once they come into contact, open the hand with all the fingers stretched to release the cube. In the Grab Game, the objective is to work both the muscle tension and distension on the hands and fingers (i.e., flexion and extension), unilateral and bilateral gross motor coordination, and gross manual dexterity due to the grabbing gesture. As in the Reach Game, the cubes are positioned at different heights and depths. Thus, the patient will be able to exercise, in addition to hands, the elbows, and shoulders and spatiality.

#### 4.1.5. Pinch Game (PG)

The opposition of the fingers is an exercise used in occupational therapy to recover fine motor skills. In this game, the bidigital grip is trained by performing the pincer movement through the terminal or subterminal opposition, both of which are valid. The patient must touch the index finger with the thumb from an initial position with extended fingers. When making this gesture close enough to the objective cube, this will acquire smaller size as the fingers approach until it disappears completely. As the cubes are reached, unilateral and bilateral gross motor coordination is trained, and additionally, in order to perform the specific task of this game, fine manual dexterity is required.

#### 4.1.6. Flip Game (FG)

**Figure 3:**
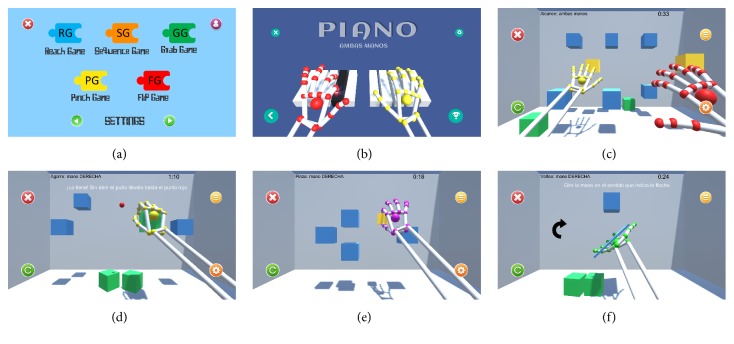
Serious Games used on protocol: (a) Games Menu, (b) Piano Game, (c) Reach Game, (d) Grab Game, (e) Pinch Game, and (f) Flip Game.

The user must situate his hand palm up over the Leap Motion device as a waiter holds a tray. A small tray filled with a cube appears in the center of the screen. The patient has to spin the palm downwards. Doing this tray rotation, the cube detaches from tray and it falls to the bottom. This game is created due to the need to exercise pronation and supination of the forearm, but also a posture control is required because it is necessary to keep the hand on the tray during the spin. In Figure 3(f), the user hand holding the small tray and an arrow to indicate the direction of rotation are shown. Once again unilateral and bilateral gross motor coordination is needed in order to reach the objects placed on the virtual space of the game. This exercise, as the previous ones, is performed individually with each hand and later the bilateral integration is carried out, taking part on the game both hands. In this case the user must coordinate the spin movement of each hand tray to drop both cubes at the same time.

### 4.2. Games Settings for Therapist

The developed games try to be as less exclusionary as possible with the target audience and the most adaptable to particularize the exercises according to each patient. In order to achieve this, a settings menu will appear in each game to adjust a set of parameters to fit the best to the capabilities and needs of the subject.

In the Piano Game, some parameters regarding the execution of the game can be changed:

- Number of repetitions: this will determine how many times the user will have to play the piano keys in order randomly and the number of sets of sequences to remember.
- Maximum time: this value will define maximum time period that is allowed without pressing a highlighted key, before a fail is registered and the game moves on to the next step. If this field is not filled, the game will wait as long as it takes until the current active key is pressed.
- Number of keys to remember during each sequence.

Also, the visual appearance of the Piano Game can be modified making use of a series of sliders to accommodate it to each patient:

- Hands' height: this is the height at which the user feels comfortable (within the Leap Motion's detection area) to complete the exercises with the hands in the air over the device. Once the patient meets the right position, the virtual hands must be placed, making use of the corresponding slider, at a height from which the keys can be pressed by only bringing down each finger.
- Distance between keys: this distance not only must be adjusted so the patient executes comfortably the exercises, but also it will define the dissociation degree between fingers.
- Key thickness: this variable establishes how much surface each key will have, thus the area that the user can touch to press them.
- Pressing height: while using the settings menu, a thin colored layer appears under the keys. When the keys are pressed and lowered until they make contact with this layer, a musical note is played, as it happens when playing a real piano. The pertinent slider can be regulated to set how much distance the key must move down to give the pressing action as valid and move on to the next one.

On the other hand, the rest of the games (RG, SG, GG, PG, and FG) can also be adjusted at performance and appearance levels:

- Number of cubes: the number of cubes shown on the screen is equivalent to the number of repetitions of each task, because the game will be completed when the exercise has been performed on each cube and all of them have fallen down to the virtual floor. In the case of bimanual exercises, the number of cubes will be double in order to match the same number of repetitions as in unilateral exercises, because each task will be executed on two cubes at a time (one target object with each hand).
- Size of the cubes: it can be chosen between small, medium, and big. The therapist can choose among them with a view to the level of difficulty.
- Depth scenario: it can be selected, depending on the protocol exercising of the patient, if the cubes appear at the same plane or at different depth distances. If it is decided to use deepness in the game, the patient will have to visually make depth discrimination and then flex and stretch the elbow to find the correct distance at which the cube is situated.
- Static or motion cubes: cubes can be arranged at a fixed position in the screen or in motion, increasing the level of difficulty. In this last case, the speed of the movement can also be chosen.
- Number of cubes to remember during the Sequence Game.
- With which finger or fingers it is valid to touch the cubes during the Reach Game: it can be selected between any combination of fingers, according to what is most appropriate for the patient's exercising. The target will be considered as reached just when it is touched with the virtual fingers which have been indicated in the settings.
- Fist closing and opening degree in the Grab Game: since not all the users have the same physical condition, a patient can find it more or less difficult to perform the grabbing gesture depending on his pathology. For this reason, the therapist is able to set up the Grab Game to be played by both a healthy user and someone who cannot close the fist completely, validating a closure degree appropriate to the user's condition (representing “0” the hand completely open and “1” totally closed). It can also be modified according to the patient's progress or to the level of difficulty of each session.
- Hand's spin in the Flip Game: when it comes to carry out the pronosupination task, the turning angle that the hand must turn during the game can be set. The values for the pronation and the supination exercises can be different between them.

These settings must be fixed before the game begins, but they can also be accessed during the exercises by pressing the settings button. This data will be registered in the user's CSV file in order to be contemplated in the patient's evaluation, but also it is useful to have them noted down in case if the exercises should be repeated under the same conditions. Although these options are available for the games, for the protocol established for the study of the Serious Games on patients with PD, it has been decided to maintain the same conditions for all the subjects and during all the sessions, so the data analysis according to patients and sessions was comparable.

### 4.3. Clinical Aspects Covered

These video games are focused on training different movements associated with daily activities. But in addition to the physical rehabilitation that is executed during each exercise and that were detailed before by each game, it has been noticed that all of them act at the same time at a cognitive and perceptive level.  Table 1 summarizes the clinical aspects.

Relative to the cognitive aspect the following features are present during the games:

- Sustained and divided attention: users must be concentrated and follow the instructions that the game will give through text, images, and voice, all of them intending to facilitate the comprehension of the exercises.
- Hand avatar: it is important that users, while playing, are able to identify and locate their virtual hands with respect to the other objects represented on screen.
- Sequencing and short-term memory: during the games that include sequence memorization, users must remember the order in which the game has shown the sequence and replicate it just after it finishes.
- Laterality: all the games take advantage of all the space that appears on the screen. The patient must be able to distinguish between the images that appear on the left, center, and right side of the screen. In unilateral exercises, the subject must reach the indicated object with the hand that corresponds on that turn, and in bilateral exercises the user must use each hand for the objects that appear on each side, respectively (i.e., objects on the left side of the screen must be reached by the left hand and vice versa).
- Executive function: it involves some cognitive processes, such as planning, organizing, or problem-solving that are required to properly perform the exercises, according to instructions the patients are given.

Regarding the perceptive factor, these video games contribute to the visuoperceptive coordination that integrate the movements of the hands and eyes and turn out to be vital in the activities performed day by day. A figure-background discrimination to hit the correct object, color discrimination which indicates targets, hits, and fails, and depth discrimination in order to find the correct position of the object to be reached is also required.

In order to compare the dexterity of each hand and its respective evolution, the exercises will be done unilaterally first with the hand less or none affected and then with the most affected. Following, in all games except in the Sequence Game, the same exercise will be performed bilaterally requiring the involvement of both hands and thus training the bimanual coordination.

### 4.4. Outcomes Storage

Rehabilitation with video games is currently intended to serve as a strong complementary tool to the traditional rehabilitation therapies. The inclusion of motion capture systems in the clinical activity provides the capability of automating some activities such as data gathering [36] and offers accurate information about the human skeleton, its joints, and their respective movements to be analyzed later by the therapist. In each one of the games created, the main variable that is recorded is the time. The partial and total times that the patient spends in each exercise are stored in a CSV file that can be easily imported into Excel, simplifying the evaluation of the results and the progress of the patients by the therapist. The user will have to fill in his details: name and surname, session number, most affected hand, and reason for the rehabilitation. This information will be stored in a CSV file named after the user's name so the results are always collected in the same file to make each patient's analysis easier. On the one hand, in the Piano Game the time that the user dedicates to press each of the keys is registered and, based on them, the average of the time spend with each finger of each hand is recorded at the end of the game, facilitating an immediate comparison between each fingers and both hands performances. On the other hand, in the rest of the games (i.e., RG, SG, GG, PG, and FG) the data recorded in the file is the time that the user takes to perform the corresponding task on each cube and the global time destined to play with each hand or both. In addition, in the Grab Game, the average degree of closure of the user's hand is computed (with “0” being the hand completely open and “1” being totally closed) and the game saves this data for its evaluation.

## 5. Feasibility Study

To evaluate the feasibility of the use of the LMC as the main capture device in a rehabilitation process, a pilot study was carried out at Asociación de Pacientes con Parkinson (APARKAN) in Alcorcón (Madrid). The main goal of the study was to validate the effectiveness of the proposed games in people in a mildmoderated stage of the PD. The pilot therapy was designed to improve the muscular strength, coordination, fine motor skills, and functionality of the upper limb in people with PD. Besides, one part of the study was focused on gathering the opinion of the participants, related to the satisfaction and the degree of adherence of them, in order to evaluate the usability of the system.

The present study obtained the favorable report from the Ethical Committee of Clinical Research of the King Juan Carlos University.

### 5.1. Pilot Trial Design

#### 5.1.1. Participants

Five individuals with PD were chosen by medical professionals to participate in this study. Participants were selected according to the following inclusion criteria: subjects with PD who met the modified diagnostic criteria of the Brain Bank of the United Kingdom; subjects in stages II, III, and IV of Hoehn & Yahr scale; sex: men and women; stable or slightly fluctuating motor response to pharmacological treatment; not having received at the time of the study a specific treatment of rehabilitation of the upper limbs; signature of informed consent form.

The exclusion criteria were diagnosis of other diseases or serious injuries that limited occupational performance; patients with other types of parkinsonism than PD; cognitive impairment affecting the language comprehension ability to follow the instructions of the study evaluation tools; refusal to participate in the study; subjects in the evolutionary stage I or V of the Hoehn & Yahr scale; visual alterations not correctable with ocular devices.

Demographic data and health status of participants in the study are summarized in Table 2.

#### 5.1.2. Treatment Protocol

Patients with PD improve their physical performance and activities of daily living through exercise, but there is no standardized exercise program for specific problems associated with PD [37]. Due to the flexibility and easy use mode of the SG presented in this paper, it is possible to make a treatment program to train different problems of motor function. The configuration of a specific treatment protocol can be seen as the pieces of a puzzle to be fitted together, according to the therapist criteria and the patient needs. Each piece of the puzzle corresponds to each video game (PI: Piano Game; GG: Grab Game; PG: Pinch Game; RG: Reach Game; SG: Sequence Game; and FG: Flip Game).

Considering the rehabilitation features (see previous Table 1) and the unilateral and bilateral training capability of each game, an appropriate game combination can be generated by therapist to deal with different cognitive, perceptual and motor problems.

**Figure 4:**
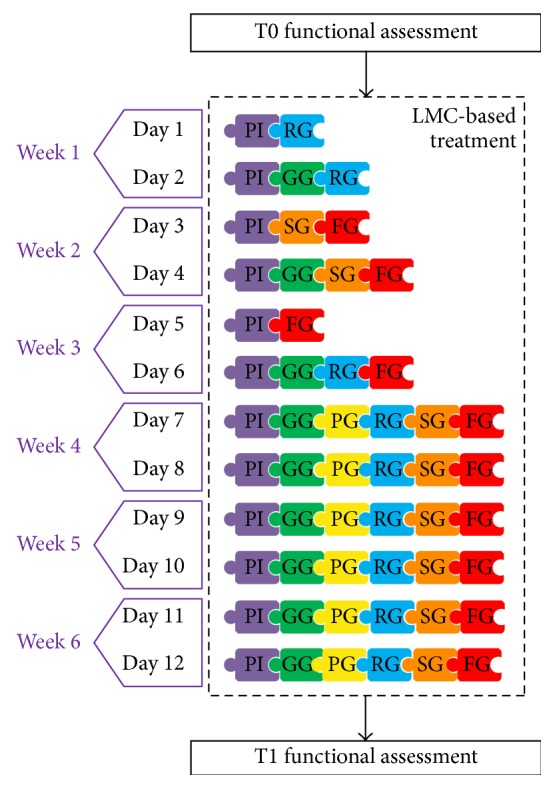
Treatment protocol scheme.

The treatment protocol followed in this study is shown in Figure 4. Training with the LMC-based video games consisted of 2 sessions a week of 30 minutes each for 6 weeks (total of 12 sessions), with the presence of a healthcare professional throughout the process. All the participants received the treatment in sedestation, with a table at the height of the middle third of the trunk and with an initial elbows flexion of 90°. In those patients who required it, manual help was provided by the therapists on the most affected side. The difficulty of the exercises was increased as well as their number as the protocol progressed, always considering the particular needs of each patient and respecting rest periods to avoid fatigue.

#### 5.1.3. Functional Assessment Method

Some standard clinical tests are used to evaluate the health condition of participants at the beginning (T0) and at the end (T1) of treatment. All participants were evaluated in the Laboratorio de Análisis del Movimiento, Biomecánica, Ergonomía y Control Motor (LAMBECOM) of the King Juan Carlos University (Madrid).

The primary outcome measure of this study was the variation between the initial (T0) and the final (T1) functional assessment, in order to quantify the effectiveness of the LMC-based training in people with PD. For that purpose, the evaluation used the following tools:

- Jamar handgrip dynamometer: it is an instrument to measure the maximum isometric strength of the hand and forearm muscles. It consists of a sealed hydraulic system with adjustable hand spacing that measures hand grip force. The strength reading can be viewed as pounds or kilograms. The dynamometer is used for testing the hand grip force and for tracking the grip strength improvements during rehabilitation.
- Box and Blocks Test (BBT): this test is used to measure unilateral gross manual dexterity in children and adults. It consists of moving the largest possible number of cubes from one compartment to another in a wooden box one by one for one minute. The results obtained in each extremity are compared. This manual procedure is automated in [36].
- Purdue pegboard test: the purpose of this test is to measure unimanual and bimanual finger and hand dexterity. Initially it was used to evaluate finger skill and manual precision in the selection of personnel who had to carry out jobs that required fine dexterity and coordination for handling small parts. At present, it is used in the clinical environment to evaluate manual dexterity. It consists of four tests: the first one consists of inserting pegs on a board with the dominant hand; the second one is to insert pegs into the board with the nondominant hand; the third one is to insert pegs with both hands; and the fourth one is to perform an assembly test using both hands alternately.

Besides, the comparative between the functional assessment results and the video games outcome will give an idea of whether the video games outcome, by itself, can be a reliable indicator of the improvement of the physical condition.

#### 5.1.4. Usability Testing

Secondary outcome measure was related to the user experience. Participants were invited to fill in a questionnaire for assessing the usability of the videogames. Questions were classified on three categories: utility, playability, and use mode. These games features were individually evaluated by each user, who expressed their opinions via a range of satisfaction scores, from −2 (strongly disagree) to +2 (strongly agree). Regarding the number of users for a proper usability assessment, five is a proper sample size for usability testing [38, 39].

### 5.2. Pilot Trial Results

#### 5.2.1. Games' Outcome

**Figure 5:**
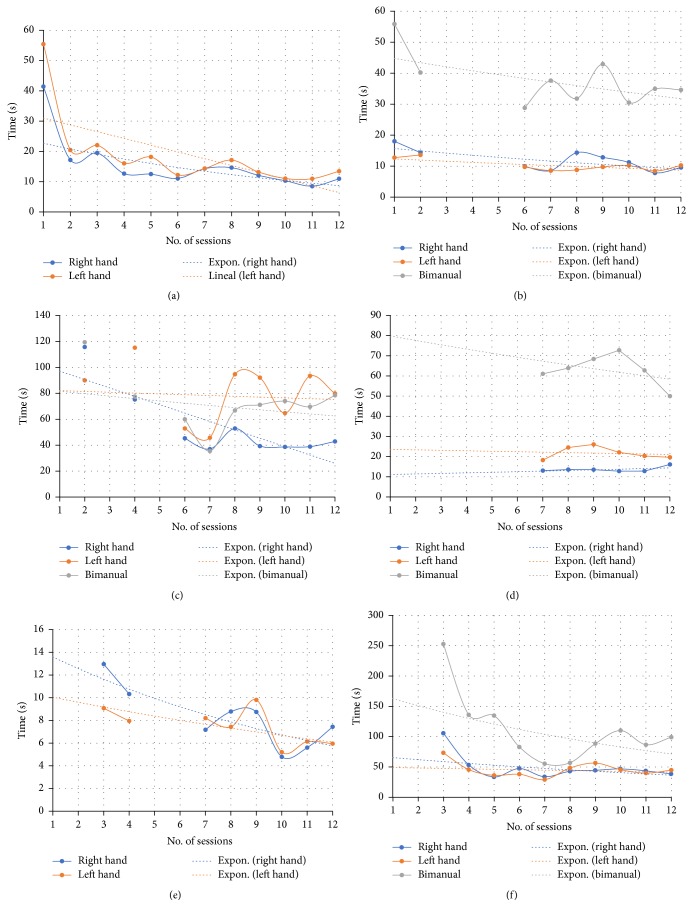
Mean of total time spent to complete the videogames tasks by sessions: (a) Piano Game, (b) Reach Game, (c) Grab Game, (d) Pinch Game, (e) Sequence Game, and (f) Flip Game.

The results obtained by the video games usage are shown in this section. On the one hand, the main outcome was the time spent to complete the exercises of each game. The average of the total time results of all users in each session is shown in Figure 5. Data are plotted according to the unilateral exercises (right or left arm) and the bilateral exercises (bimanual), including a trend line to observe the results tendency. The gaps in the curves are related to the treatment protocol, since not all the video games were used in all sessions, with the exception of the Piano Game.

In the case of Piano Game, it may be seen in Figure 5(a) that the curve corresponding to the left hand (orange line) is above the curve corresponding to the right hand (blue line). This implies that participants spent more time performing the exercises with the left hand, which is the affected hand. However, a decreasing trend is appreciated throughout the sessions.

The outcomes obtained with the Reach Game (Figure 5(b)) presents similar results for both the left and the right hand. The bimanual tasks required more time to be completed, as the curve in grey color illustrates.

In the case of Grab Game (Figure 5(c)), it can be seen that the unilateral exercises for the left hand (orange line) are very similar to the bilateral exercises (grey line). These curves are above the curve obtained with the right hand (blue line).

Data showed in Figure 5(d) are obtained by the Pinch Game. Very little variations among the values of the different sessions are observed in the case of the right and the left hand. Also, there is a remarkable variation with respect to the bimanual task that implies that the bimanual pinching task was more difficult than the unilateral one. This suggests that manual coordination was more impaired than the pinching function.

The results for the Sequence Game are shown in Figure 5(e). The measurements are very similar for both hands and it presents a clear decreasing trend. Since this video game is focused on the cognitive aspect, the results are related to a memory improvement.

Finally, in the case of the Flip Game (Figure 5(f)) the results obtained for both the right and the left hand are closely similar. Bilateral task spent more time as the line above the unilateral task shows.

**Figure 6:**
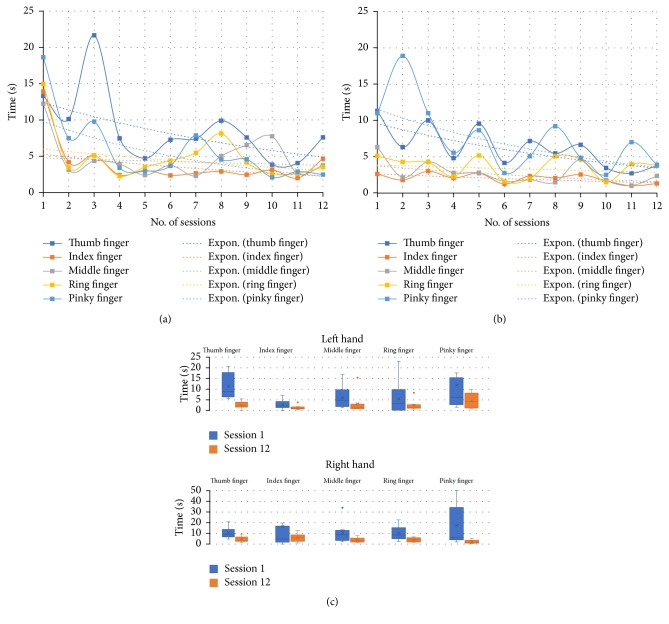
Results obtained in the Piano Game for the user 1: (a) time spent by fingers of the right hand, (b) time spent by fingers of the left hand, and (c) box plot of the partial times obtained in sessions 1 and 12, according to the left and right hand fingers.

On the other hand, other outcomes are the partial times that the patient spends to respond to a stimulus; for example, the time spent on reaching a cube in the Reach Game or pressing a key in the Piano Game. The partial time is counted from the moment the target is activated until the user “touches” it. The results obtained for user 1 in the Piano Game are shown in Figure 6. The averages of the total time spent by each finger, including unilateral and bilateral exercises, are shown in Figure 6(a) for the right hand and in Figure 6(b) for the left hand. It can be noted that the keys corresponding to both the thumb and the little finger requires more time than the rest when playing. Moreover, a box plot of the partial times obtained for the left and right hand fingers in sessions 1 and 12 is shown in Figure 6(c), to compare the user performance between the initial and final session. It can be appreciated that the data dispersion and the average in session 12 were reduced with respect to session 1. This suggests that the time of response of the fingers to a stimulus was improved in the participants.

#### 5.2.2. Functional Assessment Results

With respect to measure the efficacy of LMC-based training in PD treatment, the improvements in terms of hand grip strength, and both gross, and fine manual dexterity are shown in Tables 3, 4, and 5, respectively.

In terms of hand strength, given by the Jamar dynamometer measurement, a significant increase was obtained in four patients for the unaffected hand, while one patient (User 3) obtained a slight negative value. In the case of the affected hand, four of the participants also presented a significant improvement in grip strength, while one of the participants (User 4) obtained a remarkable negative value (see the left side figure in Table 3). The worsening in the results of user 4 can be attributed to a blow that he received in the left arm (affected side) days before the final evaluation and that caused him pain on the day of the evaluation.

Gross manual dexterity improved in all participants, according to the variation between T0 and T1 assessment in the number of the blocks that users were able to transfer by performing the BBT. As may be seen in the right side figure in Table 4, these variations in the number of blocks are very similar for both the left and the right arm of each patient, except for user 3 that is more remarkable.

The analysis of the Purdue scoring shows a general improvement by the fine manual dexterity and the eye-hand coordination (see right side figure in Table 5). It is noted that the fine manual dexterity is increased for the left hand (affected side) in all participants, while for the right hand (unaffected side) it was slightly reduced in the case of users 2 and 4. The bimanual tasks of the Purdue test require both hands coordination to be completed. Thus, the results of both the “two hands” and the “assembly” tasks revealed an improvement in the hand coordination for all participants, except for user 2 with a slight decrease and for user 5 with a more negative value in the “assembly” task.

#### 5.2.3. Usability Results

User experience by using the proposed LMC-based video games was satisfactory. Questions were classified into three categories and the results are summarized in Table 6. On the one hand, the best results were obtained in both categories “utility” and “playability,” with an average scoring of 1.68 and 1.64, respectively. Thus, the proposed video games were regarded as a useful tool to improve the independence of users in their daily living activities. The intuitive graphical design and the ease of playing were also highlighted. On the other hand, the “use mode” category obtained the worst results, with an average scoring of 0.96. Most of the participants agreed that bilateral tasks were more difficult than the unilateral ones. Bilateral exercises required more effort to be performed, and most especially in the Flip Game where some rest periods were necessary.

## 6. Discussion

The most significant feature is the flexibility of the proposed games to define a specific therapy protocol that is easy to customize to the patients particularities. Another relevant characteristic, in addition to the capability to exercise, is the potential of the proposed system as an assessment tool, taking into account the results shown in the previous section. Data for completion times (see Figure 5) has been compared with the traditional tests of manual dexterity: Purdue Pegboard Test and Box and Blocks Test. The decreasing times gathered in each session by the SG are coherent with the improvement of the physical condition of the patients, measured by the traditional tests. Although the measured times are influenced by the sensitivity of the sensor and the conditions of fatigue and mood of the users, the obtained results show a clear downward trend. This fact is consistent with the appreciation obtained by the classical metrics.

On the one hand, the improvement in the fine manual dexterity evaluated by the first part of the Purdue test presents a clear correspondence with the decrease of the average times in completing the game of Piano Game and Pinch Game. The gross manual dexterity trained mainly by the Grab Game and Piano Game has been also improved, according to the BBT results. The results obtained in bilateral execution of all the games that require bimanual coordination are consistent with the ones obtained in the second part of the Purdue test that cover this issue by means of the assembling task.

On the other hand, the fact of moving and holding the arms entails activation of the set of intrinsic and extrinsic muscles of the forearm. The training of these muscles is related to the recovery of hand strength and ability to grasp. This training of the forearm is especially enhanced by the Flip Game, thanks to pronation and supination movements. A continued and more or less intense use of the games could be related to the recovery of force measured in all the users by means of the Jamar handgrip dynamometer.

Finally, PD is extremely challenging so future technological developments could include machine learning methods to automate the rehabilitation process using LMC, by adapting the levels of difficulty and exigency of the exercises based on the subject's performance and other factors (such as fatigue, errors and success rate); serving as a complementary tool to the therapist's supervision. Additionally, there is a real challenge related to the acceptance of new technologies by the elderly population. Knowledge of the user is as important as system functionality, since without the user's cooperation, functionality may be ineffective. In this regard, a satisfaction survey was designed for gathering the impressions of participants to assess the acceptance of the proposed games, taking into account different aspects such as usability, playability, and use mode. Although, in general, the proposed video games were positively valued by participants and clinicians, the survey scores revealed the need to enhance the use mode. So, future studies should consider the effort, the difficulty, and the kind of tasks in order to facilitate the acceptance of these LMC-based video games and the integration of these technologies in a holistic rehabilitation context.

## 7. Conclusions

Despite the outcomes of the LMC-based video games were different among the training sessions, a clear decreasing trend is found throughout the treatment protocol. The improvement of health condition of participants was validated by the clinical assessment tools. The correlation between the decreasing trend and the increase in the health condition validates the video game outcomes as an indicator of improvement. This approach requires more trials to be consolidated, but it is encouraging. The influence of the mood of participants and the reliability of data acquisition must be considered also.

The Serious Games implemented in this work are a versatile tool in rehabilitation processes, since different functional problems can be treated according to the configuration defined by the therapist. Different treatment protocols can be created in an easy way.

Based on the user experience, the use of the LMC-based video games in the treatment of Parkinson's has been favorably accepted. The utility and playability of the games have been highlighted by the users; however there are certain exercises that have been difficult to perform and required the help of the therapist or breaks. This situation should be taken into account by the therapist to define a home treatment program.

Although the number of patients is not sufficiently representative to give a clinical validity to the obtained results, it is nevertheless convincing about the effectiveness of the use of these games for a double function, as an evaluation method as well as a complementary rehabilitation instrument; and it is also supported by the user experience.

## Figures and Tables

**Table 1:** Clinical aspects covered by the SG according to cognitive, motor, and perceptive functionality.

		Piano Game 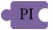	Reach Game 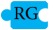	Sequence Game 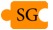	Grab Game 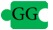	Pinch Game 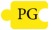	Flip Game 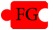
Cognitive	Sustained attention	X	X	X	X	X	X
	Divided attention	X	X	X	X	X	X
	Body image	X	X	X	X	X	X
	Sequencing	X		X			
	Short-term memory	X	X	X	X	X	X
	Problem resolution	X	X	X	X	X	X
	Executive function	X	X	X	X	X	X
	Laterality	X	X	X	X	X	X

Motor	Reaching		X	X	X	X	
	Fine motor unilateral and bilateral coordination	X					
	Gross motor unilateral and bilateral coordination		X	X	X	X	X
	Fine manual dexterity	X				X	
	Gross manual dexterity				X		
	Movement dissociation	X					
	Pronation and supination						X
	Flexion and extension				X		

Perceptive	Visuoperceptive coordination	X	X	X	X	X	X
	Figure-background discrimination	X	X	X	X	X	X
	Color discrimination	X	X	X	X	X	X
	Depth discrimination	X	X	X	X	X	X

**Table 2:** Demographics and health status of participants.

	Age	Gender	Affectation	Side	Taking medication
User 1	72	Male	Unilateral	Left	Yes
User 2	57	Female	Unilateral	Left	Yes
User 3	54	Female	Unilateral	Left	Yes
User 4	55	Male	Unilateral	Left	Yes
User 5	45	Male	Unilateral	Left	Yes

**Table 3:** Jamar handgrip dynamometer scoring in pounds (lb).

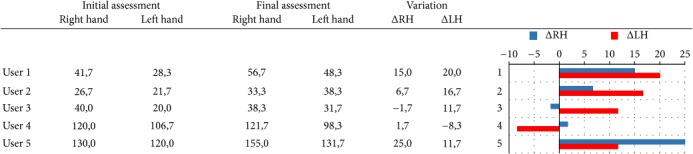

**Table 4:** Box and Blocks Test scoring.

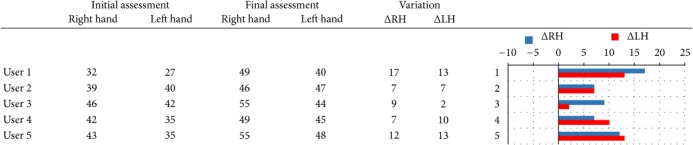

**Table 5:** Purdue Pegboard Test scoring.

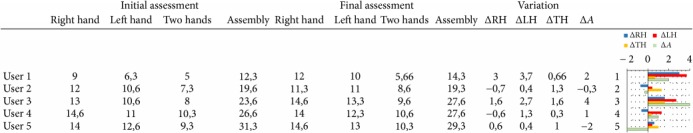

**Table 6:** Results of the usability questionnaires.

Number	Question	User 1	User 2	User 3	User 4	User 5	Mean	Mode
Utility								

Q1	Are sessions with video games more entertaining?	2	2	2	0	2	1,6	2
Q2	Have the games been interesting to you?	2	2	2	1	2	1,8	2
Q3	Do the games meet a real need?	2	2	2	1	2	1,8	2
Q4	Would you continue use the games if you could?	2	2	2	0	2	1,6	2
Q5	Would you use the games at home?	2	2	2	0	2	1,6	2

Playability								

Q6	Have the games been intuitive to play and easy to understand?	2	2	2	2	2	2	2
Q7	Have you been able to play without therapist's support?	−1	2	2	2	1	1,2	2
Q8	In case you have been helped, has the therapist's support been important?	2	2	2	0	2	1,6	2
Q9	Has the graphic design of the games been adequate (piano, cubes, etc.)?	1	2	2	2	2	1,8	2
Q10	Are the elements used in therapy sessions adequate (sensor leap motion, laptop)?	1	2	1	2	2	1,6	2

Use mode								

Q11	Have you been able to perform all the games successfully?	1	2	1	2	2	1,6	2
Q12	Have single-handed exercises been simple to perform?	2	2	2	2	2	2	2
Q13	Have the exercises with both hands been simple to perform?	−1	2	1	−1	1	0,4	−1
Q14	Have the games taken a lot of effort from you?	−1	−2	−1	−1	1	−0,8	−1
Q15	In general, the difficulty level of the games is adequate?	2	2	1	1	2	1,6	2
